# Synovial sarcoma under the lens: a case report

**DOI:** 10.11604/pamj.2024.48.154.44457

**Published:** 2024-08-05

**Authors:** Shivali Kalode, Kishor Hiwale

**Affiliations:** 1 Department of Pathology, Jawaharlal Nehru Medical College, Datta Meghe Institute of Higher Education and Research, Sawangi (Meghe), Wardha, Maharashtra, India

**Keywords:** Synovial sarcoma, swelling, spindle cells, wavy collagen, case report

## Abstract

Synovial sarcoma, an uncommon malignant tumor, can occur in various anatomical sites but mostly involves joints. It is invasive locally and tends to spread to other sites. We report a male patient, 56 years old, who had swelling above his right ankle. Soft tissue edema was observed across the lateral malleolus in imaging examinations. A synovial sarcoma was diagnosed on histopathology. A complete resection was performed and postoperative treatment with doxorubicin and cyclophosphamide was given for 2 months. The 5-year survival rate is only expected between 27-55%. Furthermore, many authors concur that synovial sarcomas are among the most frequently misdiagnosed soft tissue cancers due to their sluggish growth pattern, benign radiographic appearance, capacity to fluctuate in size, and potential to induce pain similar to that brought on by ordinary trauma. Therefore, the case emphasizes the need for additional study to identify the best diagnostic approaches for synovial sarcoma.

## Introduction

Synovial sarcoma is a relatively rare tumor representing a soft tissue sarcoma (STS) of uncertain differentiation, accounting for about 5-10% of all STS cases [1]. According to most authors, synovial sarcoma is one of the soft tissue cancers that is most frequently misdiagnosed because of its slow growth pattern, benign radiographic appearance, ability to change size, and frequent occurrence of pain resembling common injuries [2]. The majority of cases of this tumor occur in young adults and adolescents between the ages of 10 and 40. A slight male preponderance and similar incidents have been reported for all ethnic groups [3]. It has been found that there is a strong correlation between SS and the translocation of chromosomes 18 and X, resulting in the SYT-SSX fusion gene that is currently unique to SS. The sensitivity of reverse transcription-polymerase chain reaction (RPPCR) and fluorescence in situ hybridization (FISH) for diagnosing SS are reportedly about 83.8% and 80.0%, respectively, and their combined sensitivity is approximately 92.9%. Furthermore, patients with SS have a 5-year survival rate of 61%-80% and a 10-year survival rate of 10%-30% [4]. The main objective of this study is to report a case of Synovial Sarcoma.

## Patient and observation

**Patient information:** a 56-year-old male came to the Outpatient Department of Surgery at Sawangi (Meghe) Wardha with the chief complaint of swelling over his right ankle that had been gradually developing over the previous seven years and a medical history of hypertension and hyperlipidemia. According to the patient, the swelling had worsened and was especially painful on movement. There was no record of either the constitutional symptoms or the prior trauma.

**Figure 1:**
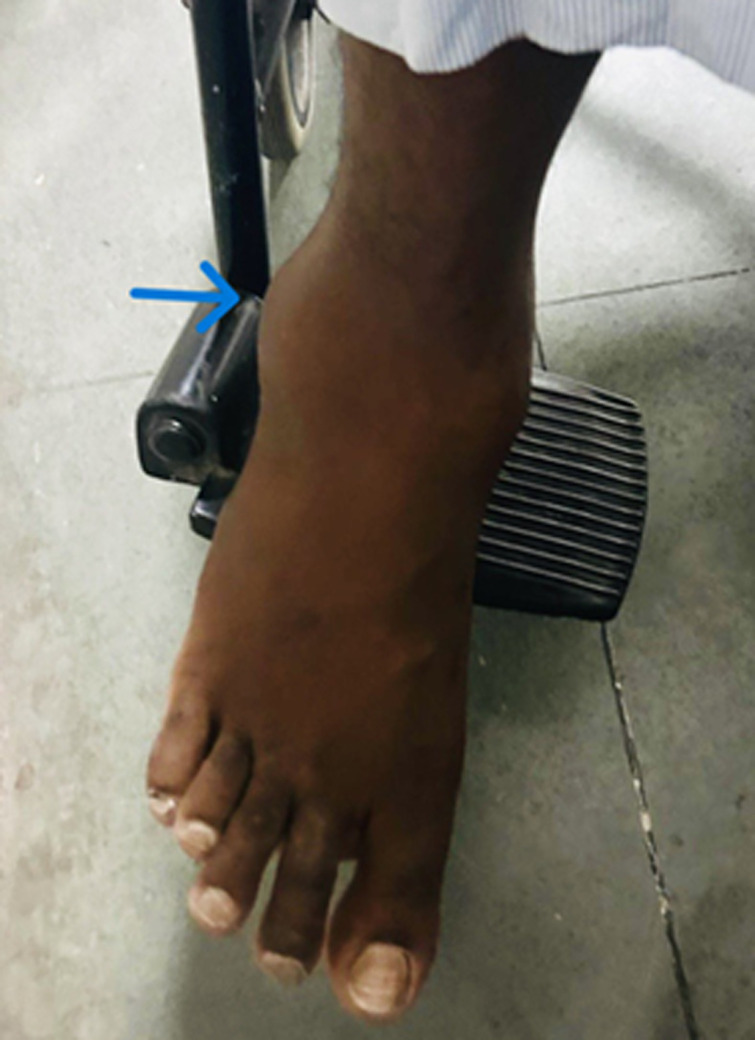
swelling over lateral malleolus of the right ankle (Arrow)

**Clinical Findings:** on physical examination, approximately 3 x 2 cm, firm to hard, nonmobile, tender bump was noticed on the lateral malleolus of the right ankle, whereas overlying skin appeared normal. No local rise of temperature or any discharge was present (Figure 1).

**Timeline of the current episode:** swelling over the right ankle for 7 years, gradually increasing to the present size.

**Figure 2:**
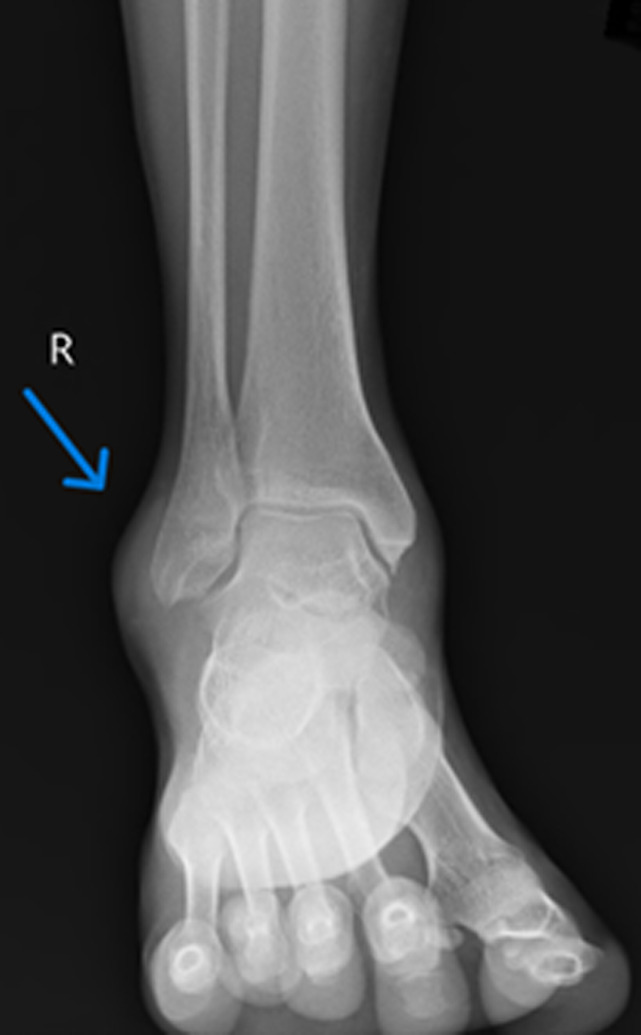
evidence of soft tissue swelling over lateral malleolus of right ankle (Arrow)

**Figure 3:**
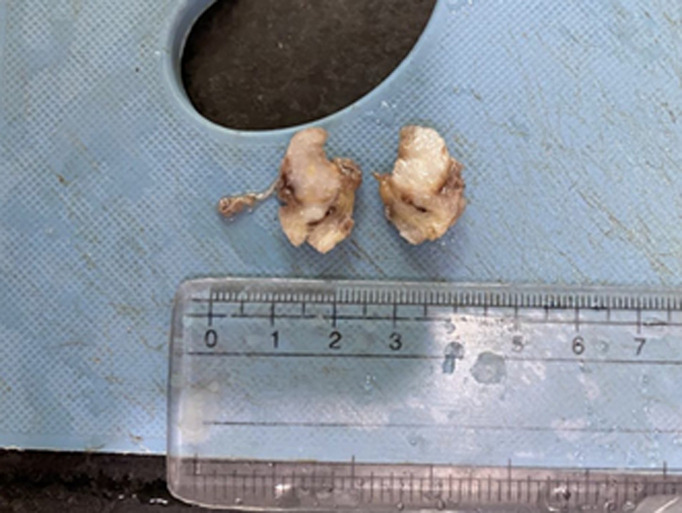
cut-section of an excised specimen of swelling

**Figure 4:**
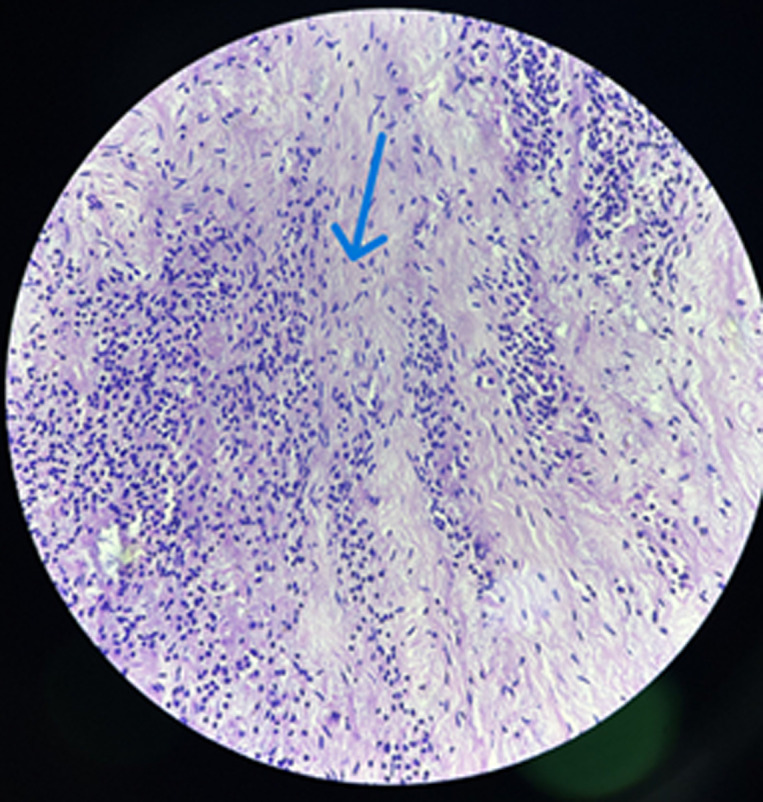
microscopic examination [H&E,40X]- the arrow showing fascicles of spindle cells, and thickened bundle of wavy collagen

**Diagnostic assessment:** a right ankle joint X-ray showed soft tissue swelling over the lateral malleolus, visualized joints and bone appeared normal with no evidence of any fracture (Figure 2). The swelling was excised after the report, and the specimen was sent for histopathological diagnosis. Grossly, the excised specimen measuring 2 x 1.5 x 1 cm, the cut section was yellowish, firm, and homogenous (Figure 3). Microscopic examination at 40X high power view showed epithelial components surrounded by fascicles of spindle cells and a thickened bundle of wavy collagen (Figure 4).

**Diagnosis:** histopathological findings confirmed the diagnosis of synovial sarcoma.

**Therapeutic interventions:** en bloc excision of the tumor was performed, followed by treatment with doxorubicin and cyclophosphamide for 2 months.

**Follow-up and outcome of interventions:** following two months of postoperative treatment with doxorubicin and cyclophosphamide, no metastases were identified in this patient.

**Patient perspective:** the patient and relative were initially worried about the diagnosis but took proper complete treatment. They were satisfied with the treatment and the patient´s recovery.

**Informed consent:** the patient gave written informed consent so that this case report and any related photos could be published.

## Discussion

A rare soft tissue tumor, synovial sarcoma (SS) accounts for 5-10% of soft tissue sarcomas and fewer than 1% of all malignancies. Synovial sarcoma is an unusual, aggressive tumor that mostly affects young adults and adolescents and has an uncertain etiology. Histopathologically, SS can take many different forms. It can be monophasic, meaning that spindle cells make up all the cells, or biphasic, meaning that both epithelial and spindle cell components are present. About 30% of patients might develop tumor calcification, which is easily detectable on standard radiography or computerized tomography [5]. En bloc excision of the tumor with distinct margins is the primary therapeutic method for synovial sarcoma. Nevertheless, cautious dissection is necessary to protect neurovascular structures when the tumor is close to them. For damaged vessels, vascular resection and reconstruction should be taken into consideration [6]. The prognosis for synovial sarcoma is not good; patients under the age of 20 have an estimated 90% chance of survival after 10 years, whereas individuals over 40 have just 25%. Its uncommon symptoms, insidious position, and low prevalence make misinterpretation and delayed diagnosis. An improved prognosis could result from early diagnosis and treatment [7].

## Conclusion

Synovial sarcoma is a rare malignancy that can be misdiagnosed, resulting in a delay in treatment. Therefore, one dealing with soft tissue tumors of upper and lower limb pathology should keep synovial sarcoma as a differential diagnosis. This in turn can reduce the chances of metastasis and increase the survival rate of the patient. Moreover, this case report contributes to other studies and articles for accurate diagnosis and management.
